# Improving cognition in severe mental illness by combining cognitive remediation and transcranial direct current stimulation: study protocol for a pragmatic randomized controlled pilot trial (HEADDSET)

**DOI:** 10.1186/s13063-021-05230-8

**Published:** 2021-04-13

**Authors:** Anika Poppe, Leonie Bais, Daniëlle van Duin, Branislava Ćurčić-Blake, Gerdina Hendrika Maria Pijnenborg, Lisette van der Meer

**Affiliations:** 1 Department of Rehabilitation, Lentis Psychiatric Institute, Lagerhout E35, 9741 KE Zuidlaren, The Netherlands; 2 Department of Clinical and Developmental Neuropsychology, University of Groningen, Grote Kruisstraat 2/1, 9712 TS Groningen, The Netherlands; 3 Trimbos Institute, Utrecht, The Netherlands; 4 Phrenos Center of Expertise, Utrecht, The Netherlands; 5 Department of Biomedical Sciences of Cells and Systems, Cognitive Neuroscience Center, University of Groningen, University Medical Center Groningen, Groningen, The Netherlands; 6 Department of Psychotic Disorders, GGZ Drenthe, Assen, The Netherlands

**Keywords:** Cognitive remediation training, Transcranial direct current stimulation, Social and functional recovery, Severe mental illness, Deinstitutionalization, Randomized controlled trial

## Abstract

**Background:**

A fundamental challenge for many people with severe mental illness (SMI) is how to deal with cognitive impairments. Cognitive impairments are common in this population and limit daily functioning. Moreover, neural plasticity in people with SMI appears to be reduced, a factor that might hinder newly learned cognitive skills to sustain. The objective of this pilot trial is to investigate the effects of cognitive remediation (CR) on cognitive and daily functioning in people dependent on residential settings. In addition, transcranial direct current stimulation (tDCS) is used to promote neural plasticity. It is expected that the addition of tDCS can enhance learning and will result in longer-lasting improvements in cognitive and daily functioning.

**Methods:**

This is a pragmatic, triple-blinded, randomized, sham-controlled, pilot trial following a non-concurrent multiple baseline design with the participants serving as their own control. We will compare (1) CR to treatment as usual, (2) active/sham tDCS+CR to treatment as usual, and (3) active tDCS+CR to sham tDCS+CR. Clinical relevance, feasibility, and acceptability of the use of CR and tDCS will be evaluated. We will recruit 26 service users aged 18 years or older, with a SMI and dependent on residential facilities. After a 16-week waiting period (treatment as usual), which will serve as a within-subject control condition, participants will be randomized to 16 weeks of twice weekly CR combined with active (*N* = 13) or sham tDCS (N = 13). Cognitive, functional, and clinical outcome assessments will be performed at baseline, after the control (waiting) period, directly after treatment, and 6-months post-treatment.

**Discussion:**

The addition of cognitive interventions to treatment as usual may lead to long-lasting improvements in the cognitive and daily functioning of service users dependent on residential facilities. This pilot trial will evaluate whether CR on its own or in combination with tDCS can be a clinically relevant addition to further enhance recovery. In case the results indicate that cognitive performance can be improved with CR, and whether or not tDCS will lead to additional improvement, this pilot trial will be extended to a large randomized multicenter study.

**Trial registration:**

Dutch Trial Registry NL7954. Prospectively registered on August 12, 2019.

## Administrative information

The order of the items has been modified to group similar items (see http://www.equator-network.org/reporting-guidelines/spirit-2013-statement-defining-standard-protocol-items-for-clinical-trials/).

**Table:** 

Title {1}	Improving Cognition in Severe Mental Illness by Combining Cognitive Remediation and Transcranial Direct Current Stimulation: Study Protocol for a Pragmatic Randomized Controlled Pilot Trial (HEADDSET)
Trial registration {2a and 2b}	Dutch Trial Registry (NL7954, www.trialregister.nl/trial/7954). Registered on 12 August 2019
Protocol version {3}	Version 2; 24.03.2021
Funding {4}	Stichting tot Steun VCVGZ, grant number 264
Author details {5a}	^1^ Department of Rehabilitation, Lentis Psychiatric Institute, Lagerhout E35, 9741 KE Zuidlaren, The Netherlands. ^2^ Department of Clinical and Developmental Neuropsychology, University of Groningen, Grote Kruisstraat 2/1, 9712 TS Groningen, The Netherlands. ^3^ Trimbos Institute, Utrecht, The Netherlands. ^4^ Phrenos Center of Expertise, Utrecht, The Netherlands. ^5^ Department of Biomedical Sciences of Cells and Systems, Cognitive Neuroscience Center, University of Groningen, University Medical Center Groningen, Groningen, The Netherlands. ^6^ Department of Psychotic Disorders, GGZ Drenthe, Assen, The Netherlands.
Name and contact information for the trial sponsor {5b}	Karen Malta, Toernooiveld 300, 6525 EC Nijmegen, The Netherlands
Role of sponsor {5c}	The sponsor played no role in the design of the study and in writing the manuscript. The sponsor will not play a role in data collection, analysis and interpretation.

## Introduction

### Background and rationale {6a}

Severe mental illness (SMI) includes a range of psychiatric disorders characterized by severe mental, social, and vocational problems that create the need for long-term continuous care by mental health professionals [1, 2]. The majority of the SMI population is diagnosed with schizophrenia or related psychotic disorders. Other common diagnoses occurring within the SMI population are severe courses of major depressive disorder, substance-related disorder, and bipolar disorder. Approximately 7% of this group requires long-term intensive psychiatric treatment and support, which in the Dutch mental health care system is provided in a clinical setting [3]. For these service users, the illness is often characterized by a chronic course and incomplete recovery [4, 5]. They can experience problems in a variety of domains, such as persistent symptoms due to medication non-adherence [6], cognitive impairments [7], physical health [8], self-care problems [9], and psychosocial dysfunctioning [9, 10].

A fundamental challenge in the treatment of people with SMI is the improvement of daily functioning with the aim to help service users to be less dependent upon (mental) health professionals. One of the core features of SMI is  substantial cognitive impairment [11]. Research suggests that impairments in cognitive functioning are even more strongly related to dysfunction in daily life [12] and level of (mental) health care dependency [13] than psychotic symptoms (e.g., delusions and hallucinations) in people with schizophrenia. The cognitive functions that have the strongest association with “functional outcome” are executive functioning (i.e., planning, organizing, structuring), verbal long-term memory, working memory, sustained attention, and social cognition [14, 15]. For example, reduced memory and impaired executive functioning hinder the execution of everyday tasks, such as shopping, self-care, and cooking. In service users with schizophrenia, significant associations have been identified between cognitive impairments and functional outcomes in terms of the ability to solve social problems, community functioning, daily activities, and the effect of psychosocial rehabilitation programs [15]. In addition, cognitive functioning appears to be a strong predictor for occupational functioning in service users diagnosed with severe mental illness [16].

Despite the high impact of cognitive impairments on functioning, there is a paucity of effective treatments that target the improvement of daily functioning through improving cognitive functioning in people with SMI. The majority of treatments used in clinical practice are aimed and often effective at decreasing positive and depressive symptoms. However, less is known about the effectiveness in the improvement of cognitive functioning. Antipsychotics, for example, do not seem to be successful at the improvement of cognitive functioning [17]. Interventions that improve daily functioning by training underlying cognitive impairments could not only foster recovery in people with SMI but also reduce health costs.

#### Cognitive remediation

In an attempt to stimulate the cognitive and functional recovery of service users with SMI, cognitive rehabilitation interventions can be applied. Within cognitive rehabilitation interventions, two different approaches can be distinguished: (1) restorative approaches that use cognitive training tasks/programs to improve (cognitive) functioning and (2) compensatory approaches that aim to bypass cognitive functioning to improve functioning. In more recent years, cognitive rehabilitation interventions have been developed that combine compensatory and restorative techniques. Collectively, these types of interventions are termed cognitive remediation (CR) training. CR is defined as a behavioral intervention targeting cognitive deficits, using the scientific principles of learning, with the ultimate goal of improving functional outcomes (Cognitive Remediation Experts Workshop (CREW), Florence, April 2010). Three meta-analyses showed that CR positively influences the cognitive performance of people with schizophrenia in general [18, 19] and in inpatients with schizophrenia [20]. It also appeared that CR can decrease negative symptoms [21] and improve the employment and income of people with SMI [22]. Essentially, CR appears to be most effective on (social) functioning of service users, when it is combined with rehabilitation programs [18, 23]. More specifically, CR programs aimed at acquiring cognitive strategies are more effective than programs that only train separate cognitive skills [18].

The term “cognitive remediation” has been used for different approaches ranging from independent computer training to psychotherapeutic approaches, and some approaches are more promising in improving daily functioning than others (for review, see [24]). To address this issue and give more clarity about the components of CR, experts in the field identified four core elements of CR: (1) a trained cognitive remediation therapist guiding and supporting the participant, (2) adaptive cognitive exercises with multiple repetitions and feedback on the process of training rather than on the performance, (3) procedures to develop problem-solving strategies, and (4) procedures to facilitate transfer to real-world functioning [25].

Reeder and colleagues [26] have developed a CR program “Computerized Interactive Remediation of Cognition and Thinking Skills” (CIRCuiTS). This innovative digital cognitive rehabilitation method aims to improve cognitive and metacognitive skills and is specifically designed to support the generalization of newly learned skills to daily life. Additionally, this method offers the possibility to personalize the training program based on individual goals and the cognitive functioning of the service user. CIRCuiTS incorporates the four core elements of CR in that (1) the therapist supports the participant with goal setting, choosing and practicing strategies, and implementing newly learned skills in daily life, (2) participants are asked to rate the difficulty of tasks and reflect on the process of training, (3) participants are encouraged to use and practice problem-solving strategies and reflect on their usefulness, and (4) ecologically valid tasks (e.g., going to the supermarket) are used and possibilities to use learned strategies in daily activities are discussed. In a randomized controlled trial, positive effects on memory and executive functioning were demonstrated when compared to treatment as usual in service users with a diagnosis of schizophrenia [27]. However, it is not known whether people with SMI who have been admitted to residential services can also profit from CR in general and from CIRCuiTS specifically. Previous work on the effectiveness of CR in this patient group using a compensatory approach (Cognitive Adaptation Training) demonstrated that improvements in daily functioning were accompanied by subsequent improvements in cognitive functioning [28]. This leads us to conclude that it may be worthwhile to assess the effectiveness of programs that aim to improve functioning through more extensive and *direct* training of cognitive functioning in inpatients with SMI.

#### Non-invasive brain stimulation with transcranial direct current stimulation

The possible benefits of CR for people with SMI may be limited as impaired cognitive functioning in schizophrenia (about 60% of this group have a diagnosis of schizophrenia [2]) is associated with reduced neural plasticity [29]. Because of this decreased neural plasticity, fewer neural connections are activated and strengthened during CR; hence, the process of learning and sustaining new skills and strategies may require very intense and frequent training [30]. The same association is known to occur with increasing age in the general population [31]. In service users who have been admitted to residential services, their relatively high age (50+ [28];) may affect the illness-related reduction of neural plasticity which forms an additional challenge to skill acquisition and retention in this group. A possible solution to this problem may be offered by the use of non-invasive brain stimulation techniques, such as transcranial direct current stimulation (tDCS), that can promote neural plasticity.

tDCS involves the delivery of a constant, or “direct”, weak electrical current via electrode patches attached to the head. Most commonly, two electrodes are used: one anode (i.e., electrode where current enters the body) and one cathode (i.e., electrode where current exits body). The administered current alters the excitability of underlying neurons, thereby modulating spontaneous neuronal network activity through a tDCS polarity-dependent shift of resting membrane potential [32]. The intended biological effect of tDCS is a shift in neuronal excitability which facilitates ongoing processes. Since the direct stimulation of neuronal firing is absent, tDCS is considered a subthreshold stimulation technique. Previous research suggests that administering tDCS may decrease symptoms and stimulate cognitive functioning, not only in people with schizophrenia [33] but also in several other psychiatric disorders [34]. For the execution of cognitive tasks, especially the frontoparietal brain networks are of importance [35], and these networks are often affected in people with a diagnosis in the schizophrenia spectrum [36]. When tDCS is being applied to frontoparietal neural networks concurrently with their engagement in cognitive tasks of CIRCuiTS, it is expected that the plasticity of these networks will be increased, resulting in long-lasting improvements in cognitive functioning. Two pilot studies suggest that such an additive effect may indeed occur in people with schizophrenia [37, 38]. In both studies, tDCS was added to working memory training, and the investigators found greater improvements in working memory and language when active tDCS was applied compared to sham tDCS. To our knowledge, the efficacy of adding tDCS to a CR program has not yet been assessed in people with SMI or schizophrenia.

#### Trial aim

The aim of the current randomized pilot study [39] is to investigate the potential clinical relevance of cognitive remediation training in service users with SMI and to evaluate the feasibility and acceptability of adding tDCS to cognitive remediation training.

### Objectives {7}

#### Primary objective

The primary objective of this pilot trial is to assess the potential clinical relevance of a full-scale trial to investigate the effectiveness of CR training with CIRCuiTS on cognitive and daily functioning in a population of service users with severe mental illness that requires long-term intensive psychiatric treatment and support in a clinical setting. The feasibility and acceptability of CIRCuiTS will be evaluated using pre-specified criteria. Additionally, we aim to obtain preliminary data on the effects of CR on cognitive and daily functioning.

#### Secondary objective

The secondary objective of this pilot study is to assess whether adding prefrontal tDCS to CT is feasible and acceptable. Furthermore, we aim for an indication of a potential additional effect of the combined intervention on cognitive performance and daily functioning compared to CR + sham tDCS.

### Trial design {8}

**Fig. 1:**
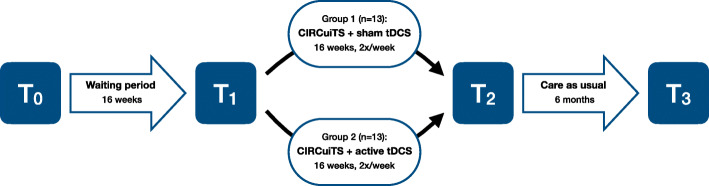
Overview of the trial design

The trial is designed as a pragmatic, randomized, controlled, pilot trial following a non-concurrent multiple baseline design with the participants serving as their own control. Participants will be randomized over two conditions: CR + sham tDCS (group 1) or CR + active tDCS (group 2) with a 1:1 allocation ratio, see Fig. 1. The trial will start with a waiting period of 16 weeks for all participants, which serves as a within-subject control condition, and is followed by the allocation of the participants to one of the treatment groups. During the next 16 weeks, the participants will receive 32 sessions of the allocated intervention. The participants will be invited back for a follow-up measurement 6 months post-treatment. In case of positive indications, we will extend the trial into a multicenter trial using the same design and recruit more participants.

## Methods: participants, interventions and outcomes

### Study setting {9}

Service users with a severe mental illness and cognitive impairments who live in one of the residential treatment or sheltered living facilities of Lentis Psychiatric Institute in the north of the Netherlands will be recruited. This institution currently provides long-term housing and treatment for approximately 500 service users with SMI.

### Eligibility criteria {10}

#### Inclusion criteria

Eligible participants are service users with an SMI, who require long-term intensive psychiatric treatment and live in a long-term housing or sheltered living facility. The criteria for SMI are based on the definitions of Delespaul et al. [2] and Parabiaghi et al. [1]: (a) a psychiatric disorder that requires care/treatment (no remission of positive, negative, and cognitive symptoms); (b) severe disabilities in social and/or societal functioning (e.g., a GAF score ≤ 50); (c) disabilities are the result of a psychiatric disorder; (d) disabilities are structural (at least 2 years); and (e) coordinated professional care is necessary to realize a treatment plan. Each participant in the study should sign informed consent. Additional criteria for inclusion are age of 18 years or older and sufficient mastery of the Dutch language.

#### Exclusion criteria

Service users will be excluded if they received CIRCuiTS previously (prior to this pilot study, we asked six patients to try out CIRCuiTS to determine whether they were able to understand the intervention and use the computer) or when the following contraindications for tDCS are present: (a) metal or electronic implants inside the skull or eye; (b) severe scalp skin lesions; (c) a history of previous seizures; (d) alcohol or drug abuse; or (e) pregnancy. Participants that show no cognitive deficits during the baseline assessment (i.e., score within their age-norm on all neuropsychological tests) will be excluded.

#### Eligibility criteria for CIRCuiTS therapists

The intervention will be delivered by trained psychologists (at least BA level) who followed the official CIRCuiTS training program (https://www.circuitstherapyinfo.com/training). All CIRCuiTS therapists will receive supervision from experienced cognitive remediation therapists once a month.

### Who will take informed consent? {26a}

The informed consent will be taken by one of the investigators. Competence to give informed consent will be judged by the service user’s treating psychologist, psychiatrist, or case manager. The project leader, an independent physician, and the rest of the researchers will be available to answer further questions. Participants are informed that the study is a randomized pilot study (i.e., that they will be allocated by chance to one of the groups) and that improvements in cognitive functioning cannot be guaranteed. As this pilot trial might be extended into a large randomized controlled trial, participants will be asked for consent to be contacted for follow-up research.

### Additional consent provisions for collection and use of participant data and biological specimens {26b}

Not applicable, participant data will not be used in ancillary studies.

### Interventions

#### Explanation for the choice of comparators {6b}

The trial will start with a baseline measurement (*T*_0_), followed by a waiting period of 16 weeks and a second baseline measurement (*T*_1_). The design has important advantages. Firstly, the waiting period serves as the control condition by which service users can act as their own control, and as such regular treatment effects can be controlled for more directly. Secondly, as no additional control group is necessary, the number of participants needed is lower than for a randomized controlled trial without a waiting period. Thirdly, as participants are their own control, all participants will receive cognitive training eventually, which is an important ethical advantage.

### Intervention description {11a}

#### Computerized Interactive Remediation of Cognition and Thinking Skills

Computerized Interactive Remediation of Cognition and Thinking Skills (CIRCuiTS) is built of tasks that are mainly aimed at the neuropsychological domains of attention, visual and verbal memory, and planning. Every task consists of eleven levels; after performing well on one level, a participant can continue to the next level. CIRCuiTS aims to improve cognition not only by training in a drill and practice approach but also by learning to use strategies and to improve meta-cognition (i.e., the ability to think and learn about one’s own cognition). Meta-cognition is trained by stimulating participants to think ahead about the tasks (how difficult will it be, how much time does it need, which strategies are useful) and reflect on their performance afterward. As such, participants are stimulated to learn about their strengths and difficulties and about which strategies can help to overcome difficulties. This will improve insight into their own cognitive abilities. To foster the generalization of new cognitive abilities and strategies to daily life, the digital environment that is used in the training is designed as a village. The tasks take place at different locations in this virtual village, such as the supermarket, train station, library, and office. The participant will choose a person (e.g., relative, friend, case manager), who will be invited to join two sessions (sessions 16 and 20). The person will learn about the goals and strategies the participant is practicing and will be encouraged to help the participant apply these strategies in daily life.

#### Transcranial direct current stimulation

In this study, tDCS will be administered using an Eldith DC stimulator (NeuroConn, Germany). The target brain area will be the left dorsolateral prefrontal cortex (DLPFC) with the anode placed at C3 and the cathode placed at Fp1 according to the international 10–20 system. The conductive rubber electrodes (5 × 5 cm; 25 cm^2^) will be fixed with conductive paste (Ten20®, Neurodiagnostic Electrode Paste, Weaver and Company, Aurora, CO, USA). For active tDCS, the current will be 2 mA and will be administered for 20 min with a fade-in/out of 30 s. For the sham tDCS, the fade-in will be identical. After 30 s the current will fade-out over 30 s. In addition to those above, a standard control pulse, with no therapeutic effect, will be frequently sent to monitor electric conductivity (~ 0.02 mA in duration of 3 s, send every 0.55 s).

We used computational modeling with the software SimNIBS [40] to infer the effects of the stimulation by the given electrode montage on the brain. With this technique, the current flow resulting from a specific electrode montage can be calculated using realistic model heads or structural MRI scans. The use of current flow modeling is recommended by experts in the field to ensure that the target region is actually targeted.

#### Treatment procedure

The duration of each training session will be 20–45 min increasing over time depending on the participants’ attention span and will be given twice weekly for 16 weeks. Participants will receive active/sham tDCS during the first 20 min of the CIRCuiTS training sessions. Evidence suggests that prolongation of treatment stimulation might not result in greater stimulation effects but can even result in smaller stimulation effects [41]. For convenience, the tDCS device will be kept in place for the whole duration of each session. The tDCS stimulation and the CIRCuiTS training will both be administered by the CIRCuiTS therapist. In every session, the participants’ mood, amount of sleep, severity of auditory hallucinations, and motivation will be assessed. Additionally, at the end of every session, the participants will be asked about sensations or side-effects of the tDCS.

The treatment procedures may be adapted at the time of the treatment to match the COVID-19 regulations and recommendations of the Dutch National Institute for Public Health and the Environment and of the Dutch Central Committee on Research Involving Human Subjects. Additionally, the guidelines for tDCS research through the COVID-19 pandemic [42] will be consulted. Necessary adaptations to the protocol will be described in the results paper and the trial register.

### Criteria for discontinuing or modifying allocated interventions {11b}

The duration of the sessions can be modified from 20 to 45 min to match the participants’ attentional capacities.

### Strategies to improve adherence to interventions {11c}

In addition to the thorough therapist training, an intervention protocol was developed by the investigators of this trial to ensure that the procedures of the intervention and strategies to improve adherence will be similar for all participants. The intervention protocol will work as a guideline for the therapist. The therapist can adapt the intervention protocol to the individual goals of the participant. To ensure adherence to the protocol, another CIRCuiTS therapist will join two sessions of all participants that consent to it and rate the sessions using a cognitive remediation training fidelity scale modified for CIRCuiTS [27]. One session from the beginning of the intervention (between week 4 and 6) and one session from the end of the treatment (between week 12 and 14) will be rated.

In addition, the intervention protocol includes procedures to engage the participant. For example, the first four sessions will take place at the participants’ rooms at the residential treatment or the sheltered housing facility. After these first sessions, the treatment will take place in a therapy room, and the therapist will pick up the participants before the start of each session. This procedure was chosen to reduce the effort the participants have to invest to successfully complete the first sessions; thereby, the therapist can increase the participants’ motivation and engagement.

### Relevant concomitant care permitted or prohibited during the trial {11d}

During the entire period of the study, all participants will receive treatment as usual according to the Dutch guidelines [43], matching international guidelines such as NICE [44], which consists of a combination of therapies and daily activities matched as much as possible to the person’s needs, goals, and wishes. The combination of therapies that a participant receives and possible changes in therapies will be recorded. Interventions that are prohibited during the trial are those that include cognitive training or non-invasive brain stimulation.

### Provisions for post-trial care {30}

Participants allocated to group 1 (CR + sham tDCS) can receive additional 32 training sessions with CR + active tDCS after the end of the complete trial.

### Outcomes {12}

#### Primary outcome measures

The feasibility of the interventions will be evaluated by procedural statistics (retention and participation rates), and the acceptability will be assessed by means of an interview with the participants. Additionally, preliminary data will be obtained for daily and cognitive functioning, and changes between assessments will be evaluated (first baseline (*T*_0_) to the second baseline (*T*_1_); second baseline (*T*_1_) to post-intervention (*T*_2_); second baseline (*T*_1_) to the 6-month follow-up (*T*_3_); and post-intervention (*T*_2_) to follow-up (*T*_3_)). Assessments will include (a) *daily functioning* measured by the Life Skills Profile (LSP [45];); (b) *cognitive functioning* measured by a set of cognitive tests selected to represent all MATRICS domains [46]: Controlled Oral Word Association Test (COWAT [47];), Modified Card Sorting Test (MCST, [48]), Digit span forward and backward [49], Rey Complex Figure Test and Recognition Trial (RCFT [50];), 15-Word Learning Task (WT-15, [51]), and Stroop Color and Word Test [52]; (c) *subjective cognitive functioning* measured by the Cognitive Failures Questionnaire (CFQ [53];) and (d) *observed cognitive functioning* measured by the Nurses’ Observation Scale of Cognitive Abilities (NOSCA [54];).

#### Secondary outcomes

##### Superior effect of active tDCS

The difference between CR + active tDCS and CR + sham tDCS in the change of daily and cognitive functioning will be assessed by the same measures described above.

##### Acceptability of tDCS

The acceptability of tDCS will be evaluated by means of an interview with the participant post-intervention.

##### Self-reported negative symptoms

Changes in self-reported negative symptoms between assessments will be measured by the Self-Evaluation of Negative Symptoms (SNS [55];).

### Participant timeline {13}

See Table 1.

**Table 1:**
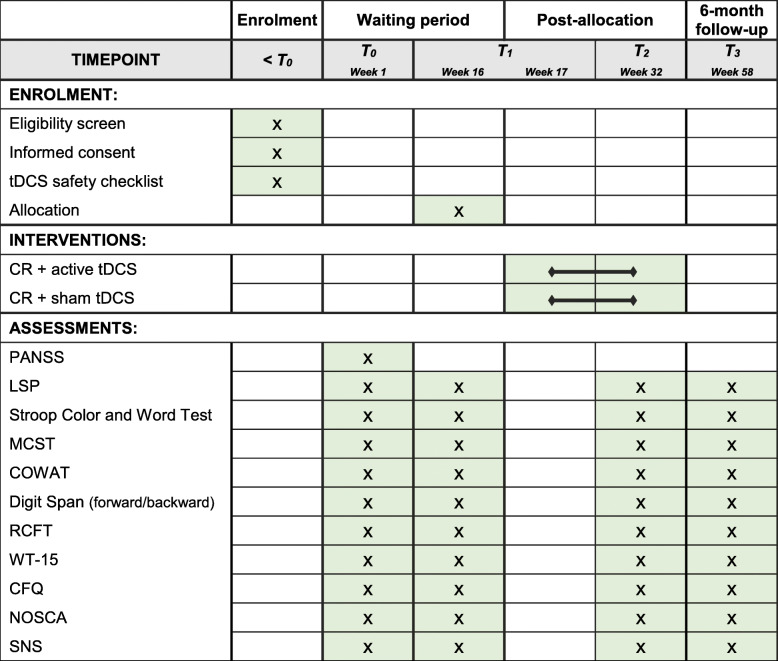
Schedule of enrolment, interventions, and assessments

*CR* cognitive remediation, *COWAT* Controlled Oral Word Association Test, *LSP* life skills profile, *MCST* Modified Card Sorting Test, *NOSCA* Nurses’ Observational Scale of Cognitive Abilities, *PANSS* The Positive and Negative Syndrome Scale, *RCFT* Rey Complex Figure Test, *SNS* Self-evaluation of Negative Symptoms, *tDCS* Transcranial direct current stimulation, *WAIS-IV* Wechsler adult intelligence scale IV, *WT-15* 15-word learning task

### Sample size {14}

For this pilot trial, the sample size is based on Whitehead et al. [56], who evaluated and compared methods for sample size calculation for pilot studies in order to minimize the recruitment of participants across pilot and main trial while maintaining the average power. The authors advise a pilot sample size of 10 participants per treatment arm if the estimated standardized effect size of the main trial is medium or large. We estimated a medium effect size based on the results of a large meta-analysis that demonstrated moderate effects of CR on cognition (effect size 0.45) and daily functioning (effect size 0.42) [18]. Taking into account a drop-out percentage of 20%, a total of 26 participants are required in order to reach a sample size of 10 participants who complete the trial per treatment arm. By considering the pilot study as a part of the main trial, this method allows to include the pilot data, if the trial is extended to a large randomized trial with a similar design.

### Recruitment {15}

The investigators will organize information meetings in the residential treatment and sheltered housing facilities to recruit participants. Additionally, nurses, psychologists, and psychiatrists will be informed about the study aims and design in information meetings and through personal communication, and they will be asked to look out for eligible participants. They will ask service users whether they give consent to be approached by the investigators of the current research project. Service users who show interest in participating will be asked to contact one of the researchers or, when desired, one of the researchers will contact them. Contact information will be given in the participant information letter. The information letter will cover all relevant information the person needs to decide whether or not to participate in the study. Service users who agree will then be informed in detail by the researchers about the goals and procedures of the experiment, both by way of an information letter and during an individual appointment. Each participant will receive a financial reimbursement of €10 per hour for the first two and the last measurement, and €15 for the third measurement (total €45).

### Assignment of interventions: allocation

#### Sequence generation {16a}

Participants will be randomly assigned to CR + sham tDCS (group 1) or CR + active tDCS (group 2) with a 1:1 allocation as per a computer-generated randomization plan using permuted blocks of random sizes. The block sizes will not be disclosed to ensure concealment.

#### Concealment mechanism {16b}

A randomization sequence will be generated by a randomization plan generator (http://randomization.com). The tDCS device is pre-programmed with codes that are linked to either real or sham stimulation. Twenty-six codes, which are printed in the manual, will be selected randomly (13 for sham stimulation, 13 for real stimulation) and linked to the randomization sequence. The manual page with the codes and the randomization sequence will be kept in two separate sealed envelopes which will remain closed until the completion of the data collection.

Only the sequence of the codes will be accessible during the trial, which cannot be linked to real or sham stimulation without the randomization sequence or the manual page. When participants complete the second baseline measurement (*T*_1_), the first available code in the sequence will be coupled to the participant number.

#### Implementation {16c}

An independent researcher will generate the allocation sequence, select the tDCS codes, link them to the randomization sequence and seal the envelopes. The investigators will enroll participants, link the tDCS code to the participant number following the second baseline measurement (*T*_1_), and communicate the code to the CIRCuiTS therapist. The randomization sequence and the block sizes will not be disclosed until all participants completed the follow-up measurement (*T*_3_). Thus, randomization will be conducted without any influence of the investigators, raters, or therapists.

### Assignment of interventions: blinding

#### Who will be blinded {17a}

Using the allocation procedure described above the trial will be triple-blind to the tDCS experimental condition; the investigators, the CIRCuiTS therapist, and the participant will stay blind to the tDCS treatment condition. The CIRCuiTS therapist and participant will be asked to indicate whether the participant received either active or sham stimulation in session 10 of the intervention and at *T*_2_. The participants will be informed about the type of treatment they have received after completion of the trial. As all participants will receive CIRCuiTS, the investigators, CIRCuiTS therapist, and participants will not be blind to the CIRCuiTS experimental condition.

Research assistants (at least BA level psychologists) will be trained to administer the assessments. They will be blind to the experimental condition (both tDCS and CIRCuiTS) of the participants, as they will not receive any information regarding the aims and design of the trial, nor of the intervention. Guessing the aim of the study and the allocation group of the participant (“Experimental,” “Control,” “There is no control or experimental condition,” or “I don’t know”) after every assessment will be used to evaluate whether blinding was successful.

#### Procedure for unblinding if needed {17b}

Not applicable, as there are no circumstances during the trial under which unblinding will be permissible.

### Data collection and management

#### Plans for assessment and collection of outcomes {18a}

##### Testing procedure

Outcome measures for both groups will be assessed before (*T*_0_) and after the waiting period (*T*_1_), after completion of the treatment (*T*_2_), and 6 months post-treatment (*T*_3_). After T_2_, an interview will be conducted. All tests and measures are described below. The assessments will be administered by trained research assistants. Demographical information will be obtained from the patient file. In order to describe the clinical characteristics of the participant group at baseline, the Positive and Negative Syndrome Scale (PANSS [57];) is used. The PANSS is part of the yearly Routine Outcome Monitoring screening, which is performed by a trained nurse. Though psychiatric symptoms in service users of residential and sheltered living departments are generally stable (and for which the yearly screening data will suffice), it is possible that symptoms in some service users have fluctuated in the period between the last PANSS interview and the start of the study. To address possible fluctuations in symptoms, the treating clinician will be consulted preceding the start of the first baseline assessment. In case of changed symptomatology, an additional PANSS will be administered by a trained nurse, which will take 30 min.

#### Baseline measures

##### Demographical information

The following demographical and clinical information will be collected: year of birth, gender, nationality, level of education, level of education of parents, main diagnosis, comorbid diagnoses, age at first hospitalization, medication use, psychiatric disorders of parents and siblings, relevant medical events, neurological disorders, and alcohol and drug use.

##### Severity of clinical symptoms

The Positive and Negative Syndrome Scale (PANSS, [57]) is a semi-directive interview developed, standardized, and validated for the typological and dimensional characterization of schizophrenia. It consists of 30 items; each scored on a 7-point scale and divided between a scale for positive symptoms, a scale for negative symptoms, and a general psychopathology scale. The total PANSS scale is judged as stable, valid, and reliable [57]. The Dutch version of the PANSS is validated by Wolthaus et al. [58].

##### Expectations

As participants’ expectations may influence the cognitive performance following tDCS administration [59], participants’ expectations will be assessed prior to the start of the intervention. They will be asked if they believe (1) that the intervention can improve cognitive and daily functioning in general, and (2) that the intervention can improve their own cognitive and daily functioning.

#### Outcome measures

##### Feasibility

The feasibility will be determined by the retention and participation rates of the intervention.

1. Retention: The intervention will be considered feasible if > 60% of the sample completes the study. The threshold for indicative feasibility was determined based on the retention and participation rates in previous studies. Previous CR studies in inpatient populations with schizophrenia had retention rates of 58–92% [20, 27, 60, 61]. Previous studies that combined cognitive training and non-invasive brain stimulation in people with schizophrenia had retention rates of 58–100% [62, 63].
2. Participation rates: The intervention will be considered feasible if participants complete > 62.5% (i.e., 20 sessions) of the intervention sessions, as in the RCT of CIRCuiTS by Reeder and colleagues, the developers of CIRCuiTS judged that a minimum therapy course comprises 20 sessions [27].

##### Acceptability

The Theoretical Framework of Acceptability developed by Sekhon et al. [64] will be used to evaluate the acceptability of the intervention. Within this theoretical framework, acceptability is defined as “a multi-faceted construct that reflects the extent to which people delivering or receiving a healthcare intervention consider it to be appropriate, based on anticipated or experienced cognitive and emotional responses to the intervention” and consists of seven component constructs: Affective attitude, burden, intervention coherence, ethicality, opportunity costs, perceived effectiveness, and self-efficacy. To evaluate acceptability within the seven components of acceptability, all participants will be interviewed regarding these components following the post-intervention assessment (*T*_2_). This interview will take approximately 15 min.

##### Functional outcome measure

The Life Skills Profile (LSP [45];) is a questionnaire consisting of 39 questions that are scored on a 4-point scale (lower score indicates higher life skills). The questionnaire is developed from a positive mental health philosophy, by emphasizing “life skills” rather than “lack of life skills” and measures a range of aspects related to successful community or hospital living: (1) self-care, (2) non-turbulence, (3) social skills, (4) communication, and (5) responsibility. The LSP has shown good psychometric properties when completed by residential staff and case managers [45] and will be completed by a participants’ case manager.

##### Cognitive outcome measures

The tests of the cognitive test battery were chosen to represent the domains of the Measurement and Treatment Research to Improve Cognition in Schizophrenia (Nuechterlein et al., 2008) test battery. The administration of the MATRICS battery is not feasible in the target group due to its length. Hence, we chose tests that match the skills of the target group.

- *Speed of processing*. Controlled Oral Word Association Test (COWAT [47];) is a test for verbal fluency and speed of processing. Participants have to name as many words as possible within a time span of 60 s, beginning with a given letter. The score is the total number of words of three trials. This test will take 10 min.
- *Attention and working memory.* Digit Span forward (WAIS-IV, [49]) is a test for immediate auditory attention. The test leader reads sequences of digits out loud, gradually increasing the digit span. The participants are asked to repeat the digits in the same order. The score is determined as the number of correctly recalled sequences. Digit Span backward (WAIS-IV [49];) is a test for working memory. It is executed similarly as the previous test; however, the digits have to be repeated in reverse order. Both tests will take 5 min.
- *Visual memory.* The Rey Complex Figure Test (RCFT) is a measure for visual memory [50]. The test leader presents a complex figure that participants are asked to copy (copy trial). Thirty minutes later, the participants are asked to draw the complex figure from memory (recall trial). This test will take approximately 5 min.
- *Verbal memory.* The 15-word learning task, immediate memory (WT-15 [51];) is a test for verbal memory. The test leader reads 15 words out loud and participants are asked to recall as many words as possible. This procedure is repeated five times. The score is determined as the total number of reproduced words (max. 75). Additionally, after continuing with the test battery for 15 min, the participant will be asked to name as many words as they can remember (free recall). This test will take approximately 20 min to complete.
- *Reasoning and problem solving.* The Stroop Color and Word Test [52] is used to test speed of processing, inhibition, cognitive flexibility, and cognitive control. The participants first perform a basic task, which is reading the names of colors. The performance on this task is compared with the performance on a task in which the ink color of incongruent color words has to be named. Hence, a habitual response needs to be suppressed in support of an unusual one. The increase in time taken to perform the latter task compared with the basic task is referred to as “the Stroop interference effect” and provides a general measure of executive functioning. The total duration of the Stroop Task will be approximately 5 min.
- *Reasoning and problem solving.* Modified Card Sorting Test (MCST [48];) is a test for cognitive flexibility and reasoning. The test leader presents four stimulus cards with different shapes and colors. Participants receive a pile of 48 cards and are asked to lay down the cards one by one on the four presented cards, following a rule that the participant has to discover. The score is determined as the number of correct categories and the number of errors (e.g., perseverations). The MSCT is a simplified version of the Wisconsin Card Sorting Test and is easier to understand for people with a disorder and will take approximately 10 min.
- *Subjective cognitive functioning.* The Cognitive Failure Questionnaire (CFQ) is a questionnaire to measure subjective cognitive functioning [53, 65]. The scale includes 25 questions that represent the cognitive subdomains of attention and memory. The questionnaire can be completed by the participants and takes approximately 5 min.
- *Observed cognitive functioning.* The Nurses’ Observation Scale of Cognitive Abilities (NOSCA [54];) is a behavioral rating scale to examine cognitive abilities. The scale includes 39 items that are scored on a 4-point scale, and it comprises eight subscales: attention, perception, memory, orientation, thoughts, language, and praxis. The NOSCA will be completed by the participants’ case manager. This scale will take approximately 10 min to complete.

##### Clinical outcome measure

Participant’s negative symptoms at baseline will be assessed with the Self-Evaluation of Negative Symptoms (SNS [55];). This self-report questionnaire contains 20 items covering the five domains of negative symptoms (social withdrawal, diminished emotional range, avolition, anhedonia, and alogia) and can be scored on a three-point Likert scale, based on the feelings during the previous week. The administration of the SNS will take approximately 5 min. The questionnaire is designed and validated by Dollfus et al. [55] and has shown good psychometric properties and is well-tolerated by service users. The English version of the questionnaire is translated into Dutch by the investigators of this study, back-translated to French by a native French speaker, and approved by Prof. Dr. S. Dollfus.

### Plans to promote participant retention and complete follow-up {18b}

As the intervention has a duration of 4 months and takes place twice weekly, it is possible that participants will miss sessions, for example, because of illness, vacation, or important (medical) appointments. In the RCT of CIRCuiTS by Reeder and colleagues, the developers stated that a minimum therapy course comprises 20 sessions, which includes both sessions with the therapist and independent homework sessions [27]. Accordingly, participants will be considered completers, if they complete at least 20 sessions, and non-completers, if they complete less than 20 sessions. As we are interested in the feasibility and acceptability of the intervention, participants who discontinue the intervention protocol will be encouraged to take part in the intervention evaluation interview at the moment of discontinuation. Participants who drop out or deviate from the intervention protocol (i.e., complete less than 20 sessions) will be invited back for the assessments at *T*_2_ and *T*_3_.

### Data management {19}

In the trial, all data will be entered digitally at Lentis Psychiatric Institute. Original study forms will be saved in files and stored at the study site. Participant files are to be stored in numerical order and stored in a secure place for a period of 15 years. The data entry screens will resemble the paper forms. Data integrity will be enforced through range checks and consistency checks. Modifications to data written to the database will be documented in a separate file. A complete backup of the database will be performed once a month. A data management plan will be developed to summarize all procedures of data management.

### Confidentiality {27}

All data received from participants will be processed in a strictly confidential manner. Except for the written consent and the proof of payment, no other questionnaires or documents will contain any personal data (name, address, date of birth) of the participants, but will be attributed by a code. The participant code can only be traced back by accessing a protected key file. The password to the key file will only be shared with the researchers who are involved in the data collection. Researchers, other than those immediately involved in the data collection and in the intervention, only have access to fully anonymous files, which cannot be traced back to a specific individual. Data used for publication are also completely anonymous. All handling of personal data will comply with the Dutch General Data Protection Regulation (Dutch: Algemene Verordening Gegevensbescherming (AVG)).

### Plans for collection, laboratory evaluation, and storage of biological specimens for genetic or molecular analysis in this trial/future use {33}

Not applicable, no samples collected.

### Statistical methods

#### Statistical methods for primary and secondary outcomes {20a}

##### Acceptability

The acquired data from the Intervention Evaluation Interview will be analyzed qualitatively using the qualitative data analysis and research software ATLAS-ti.

##### Cognitive and functional outcome measures

For all the tests, sum scores will be calculated and related to normative data for each individual. Averages and standard deviations will be calculated per training condition. Subsequently, all variables will be checked for normality. To measure demographical and baseline differences between the two groups, an independent sample *t*-test will be performed.

##### CR vs. treatment as usual

The mean differences in outcomes of both groups combined will be compared to the waitlist period using multilevel modeling to assess the effect of CR over treatment as usual. A two-level model will be built with subject (level 2) and time point of assessment (level 1) entered as levels. The significance of the fixed regression effects is tested using the appropriate *t-*test (*α* = .05).

##### CR + active tDCS vs. CR + sham tDCS

The mean differences in outcomes between groups 1 and 2 will be compared using multilevel modeling to assess whether active tDCS in combination with CR is superior in improving cognitive functioning and daily functioning over CR + sham tDCS. A two-level model will be built with subject (level 1) and time point of assessment (level 2).

#### Interim analyses {21b}

Not applicable; no interim analyses will be performed.

#### Methods for additional analyses (e.g., subgroup analyses) {20b}

Not applicable; there are no additional analyses planned because of the small sample size.

#### Methods in analysis to handle protocol non-adherence and any statistical methods to handle missing data {20c}

Data will be analyzed according to intention-to-treat principles. The extent of missing data will be explored in the outcomes and predictors of missingness will be examined.

#### Plans to give access to the full protocol, participant-level data, and statistical code {31c}

This document is the full protocol. Participant-level data and statistical code will be available from the corresponding author on reasonable request.

### Oversight and monitoring

#### Composition of the coordinating center and trial steering committee {5d}

The trial is coordinated and steered by the investigators of this study. The project management group (AP, LB, and LM) provides the day-to-day support for this trial. AP is responsible for the identification of potential participants, recruitment, taking consent, coordinating data collection, overseeing adherence to the study protocol, and keeping the participants involved during the waiting period (see “Discussion”). LB and LM supervise AP in all of these steps and will monitor the progress and the conduct of the study. DD and GM provide supervision for the cognitive remediation training. The progress and conduct of the study and any identified or planned deviations from the study protocol will be discussed with BC and GM. All authors will contribute to writing the manuscript describing the results of the trial.

#### Composition of the data monitoring committee, its role, and reporting structure {21a}

There will not be a data monitoring committee as the treatments under consideration are regarded as safe methods.

#### Adverse event reporting and harms {22}

When applied following established safety protocols, tDCS is regarded as a safe method of brain stimulation, causing no apparent short- or long-term harm [42]. The effects and risks of tDCS are limited by safety protocols that limit the applied current, duration, and frequency of stimulation sessions [66]. The parameters used in this trial are within the safety guidelines; therefore, adverse effects are minimized. At the end of every tDCS session, the participants will be asked whether they experienced any adverse effects (e.g., headaches, itching, tingling, burning), how severe possible adverse effects were (from very mild to severe), and whether they relate the adverse effect to the stimulation as recommended by Aparicio et al. [67].

All adverse events, whether or not considered related to the cognitive remediation training or tDCS, that are reported by the participant or observed by the investigators or health professionals, will be recorded from the time a participant consents to join the trial until the last trial visit. The adverse events will be assessed for seriousness and will be reported to relevant regulatory bodies.

#### Frequency and plans for auditing trial conduct {23}

No formal, external audit will be conducted. The project management group will meet bi-weekly to review the trial conduct, enrollment rates, and to oversee and conduct the study in general. In monthly meetings of the project management group, BC, and GM, the conduct and progress of the study will be discussed.

#### Plans for communicating important protocol amendments to relevant parties (e.g., trial participants, ethical committees) {25}

Important protocol modifications which may have an impact on the conduct of the study, the potential benefit for the participant, or may affect participant safety will be communicated to the trial participants, trial registries, the Ethical Committee, and the sponsor. Minor corrections or clarifications of the protocol that have no effect on the way the study is conducted will be documented in a list of amendments.

### Dissemination plans {31a}

The results of this pilot study will be publicly disclosed. Presentations of the results will be given at (inter-)national conferences. Moreover, the results will be published in an international scientific journal, as well as in a Dutch journal to reach more clinicians in the field.

## Discussion

Impaired cognitive functioning in SMI is related to a higher level of dependency on mental health care [13], as well as lower levels of daily [12], community [15], and occupational functioning [16]. Hence, interventions that aim to improve cognitive functioning are needed to foster recovery. The presented trial will help to elucidate whether the addition of innovative cognitive enhancement interventions to treatment as usual can lead to clinically relevant improvements in the daily functioning of service users dependent on residential facilities and whether these improvements can be retained beyond the treatment period.

This study differs from previous trials in that it investigates the effects, the feasibility, and acceptability of a CR program that incorporates all four core elements of CR as defined by Bowie et al. [25], in a population with severe mental illness dependent on long-term support in a clinical or sheltered setting, which has not been done before. While some trials investigated the efficacy of cognitive training in inpatients with psychosis [20], none of the administered programs included all four core components of CR. These cognitive training approaches in inpatients with schizophrenia were effective in improving cognitive functioning, but not daily functioning. As CR has shown the potential to provide benefits in both cognitive and daily functioning in people with schizophrenia [18, 19], these CR programs may also enhance daily functioning in people with SMI.

Additionally, this trial combines cognitive remediation with non-invasive brain stimulation. Previous trials focused mainly on combining tDCS with cognitive training approaches that were based on an independent, repetitive, drill, and practice approach. The improvement achieved by this independent approach is not only limited to cognitive functioning when used as stand-alone treatment [24] but also when used in combination with non-invasive brain stimulation (Poppe et al., in preparation). To our knowledge, this trial is the first to combine tDCS with CR program in a psychiatric population.

In the presented trial, a multiple baseline randomized controlled design is used. This trial design has several advantages. Firstly, prior research has demonstrated the efficacy of the CR program used in this trial [26, 27]. In the multiple baseline design, every participant can be exposed to the intervention. The use of this design is, therefore, more ethical than a traditional randomized controlled trial, in which individuals in control groups are not exposed to the intervention of interest.

Secondly, the 4-month waiting period serves as a control condition and allows investigating the effects within individuals. A within-subject analysis is more informative than group comparisons in this trial, as individual differences in various domains can influence the efficacy of the treatment: for example, the cognitive deficits experienced, which may occur within different cognitive domains [68]; and physiological factors related to the current flow of tDCS such as skull thickness, subcutaneous fat levels, cerebrospinal fluid density, and cortical surface topography [69]. Using within-subject analyses allows determining whether the intervention is superior to treatment as usual for each individual. If the intervention appears to be more effective for some individuals than for others, then the differences between those individuals can be further investigated.

However, the use of a multiple baseline design has some disadvantages and challenges. Firstly, the assessments happen over a period of time and the control condition will, on average, contribute data from an earlier calendar time. Therefore, time effects could influence the results. However, psychiatric symptoms and cognitive impairments are generally stable in the targeted inpatient population, which minimizes the risk of time effects. A second challenge arising due to the presence of a waiting period is that participants might become less engaged during the waiting period and drop out before the intervention has started. Thus, it will be important to keep regular contact with the participants during the waiting period. The therapist will contact all participants once a month, to keep the engagement process as similar as possible across the whole group. While keeping the participants engaged may be a challenge, the contact moments allow building up a therapeutic relationship with the participant which could relate to higher motivation during the intervention. Thirdly, as all participants receive CR, the investigators, the therapist, and the participants are not blind to whether or not participants receive CR. To control for possible influences of this disadvantage to the assessments, assessors will be kept blind to the intervention conditions by not disclosing the study aims and design to them.

Another challenge in this trial is the evaluation of the superiority of adding active tDCS to the cognitive remediation over sham tDCS. Active tDCS will be added to the treatment of only half of the participants, and the efficacy will be analyzed in a group analysis. The chosen study design requires fewer participants than a traditional randomized controlled trial to investigate the CR efficacy given the within-subject comparison. Yet, because of the small sample size and the expectation that CR on its own can improve cognitive and daily functioning, the power to detect significant differences between the groups with respect to tDCS efficacy is low. While statistical significance may be difficult to achieve, this pilot study can at a minimum identify a trend towards superior treatment effects of combined CR and tDCS as compared to CR only. If the addition of tDCS seems to have an additive effect on CR, we aim to initiate a multicenter randomized controlled trial to further investigate the efficacy.

In summary, this pilot trial could offer new insights into the possibility of improving cognitive functioning in people with severe mental illness. The use of a waitlist control condition enables us to investigate the clinical relevance of cognitive enhancement interventions in the heterogeneous population of people with SMI. Moreover, this trial may contribute to the discovery of new interventions that foster the functional recovery of service users living in long-stay clinical facilities.

### Trial status

Recruitment is currently ongoing. Participant enrollment started in October 2020 and will continue until 26 participants are included in the study. The predicted recruitment end date is 31 December 2021.

## Abbreviations

CFQ: Cognitive Failure Questionnaire
CIRCuiTS: Computerized Individualized Remediation of Cognition and Thinking Skills
COWAT: Controlled Oral Word Association Test
CR: Cognitive remediation
CREW: Cognitive Remediation Expert Working Group
DLPFC: Dorsolateral prefrontal cortex
LSP: Life skills profile
MATRICS: Measurement and Treatment Research to Improve Cognition in Schizophrenia
MCST: Modified Card Sorting Test
NOSCA: Nurses’ Observational Scale of Cognitive Abilities
PANSS: The Positive and Negative Syndrome Scale
RCFT: Rey Complex Figure Test
SMI: Severe mental illness
SNS: Self-evaluation of Negative Symptoms
tDCS: Transcranial direct current stimulation
WAIS-IV: Wechsler adult intelligence scale IV
WT-15: 15-word learning task
